# The Mood-Improving Effect of Viewing Images of Nature and Its Neural Substrate

**DOI:** 10.3390/ijerph18105500

**Published:** 2021-05-20

**Authors:** Rikuto Yamashita, Chong Chen, Toshio Matsubara, Kosuke Hagiwara, Masato Inamura, Kohei Aga, Masako Hirotsu, Tomoe Seki, Akiyo Takao, Erika Nakagawa, Ayumi Kobayashi, Yuko Fujii, Keiko Hirata, Harumi Ikei, Yoshifumi Miyazaki, Shin Nakagawa

**Affiliations:** 1 Division of Neuropsychiatry, Department of Neuroscience, Yamaguchi University Graduate School of Medicine, Ube 755-8505, Japan; i096eb@yamaguchi-u.ac.jp (R.Y.); t-matsu@yamaguchi-u.ac.jp (T.M.); khagi@yamaguchi-u.ac.jp (K.H.); g009eb@yamaguchi-u.ac.jp (M.I.); i001eb@yamaguchi-u.ac.jp (K.A.); hirotsu@yamaguchi-u.ac.jp (M.H.); tseki@yamaguchi-u.ac.jp (T.S.); v053eb@yamaguchi-u.ac.jp (A.T.); v069eb@yamaguchi-u.ac.jp (E.N.); ayumi@yamaguchi-u.ac.jp (A.K.); yuko-f@yamaguchi-u.ac.jp (Y.F.); hiratak@yamaguchi-u.ac.jp (K.H.); snakaga@yamaguchi-u.ac.jp (S.N.); 2 Center for Environment, Health and Field Sciences, Chiba University, Chiba 277-0882, Japan; hikei@chiba-u.jp (H.I.); ymiyazaki@faculty.chiba-u.jp (Y.M.)

**Keywords:** green plants, affect, natural environment, near-infrared spectroscopy, orbitofrontal cortex, relaxation

## Abstract

It has been recently suggested that contact with nature improves mood via reducing the activity of the prefrontal cortex. However, the specific regions within the prefrontal cortex that underlie this effect remain unclear. In this study, we aimed to identify the specific regions involved in the mood-improving effect of viewing images of nature using a 52-channel functional near-infrared spectroscopy (fNIRS). Specifically, we focused on the orbitofrontal cortex (OFC) and dorsolateral prefrontal cortex (dlPFC), two regions associated with affective processing and control. In a randomized controlled crossover experiment, we assigned thirty young adults to view images of nature and built environments for three minutes each in a counterbalanced order. During image viewing, participants wore a fNIRS probe cap and had their oxyhemoglobin (oxy-Hb) measured. Immediately following each image viewing, participants indicated their mood in terms of comfortableness, relaxation, and vigor. Results showed that viewing images of nature significantly increased comfortableness and relaxation but not vigor compared to viewing images of built environments, with a large effect size. Meanwhile, the concentration of oxy-Hb in only the right OFC and none of the other regions significantly decreased while viewing the images of nature compared to built environments, with a medium effect size. We speculate that viewing images of nature improves mood by reducing the activity of or calming the OFC. Since the OFC is hyperactive in patients with depression and anxiety at rest, contact with nature might have therapeutic effects for them.

## 1. Introduction

The affective benefits of contact with nature have been extensively studied and reviewed [1,2,3,4,5]. Contact with natural environments, even briefly, increases positive affect, such as comfortableness and relaxation, decreases negative affect, such as anxiety and depression, and alleviates psychological stress. Furthermore, these benefits are not limited to contact with outdoor, “real” natural environments but also indoor, “virtual” natural environments (e.g., [6]). These benefits are not constrained to contact with nature via all the five senses; contact with nature via even a single sense, be it sight, smell, touch, sound, or taste, achieves many of the affective benefits as well (e.g., [7]). Of the five senses, the most studied one is sight. Although to a lesser extent compared to contact with real nature, viewing or visual stimulation with images of natural environments (i.e., virtual nature), in particular those featuring green plants, has been found to improve mood and increase affective wellbeing [6,8].

Recently, there is increasing interest in the neurobiological basis of the affective benefits of contact with nature [9,10,11,12,13]. A commonly employed technique for investigating neurobiological basis is functional near-infrared spectroscopy (fNIRS). A non-invasive neuroimaging technique, fNIRS is based on the observation that near-infrared light is preferentially absorbed by oxyhemoglobin (oxy-Hb) and deoxyhemoglobin (deoxy-Hb). It emits near-infrared light onto the brain surface to measure the regional cerebral blood flow in terms of the relative concentrations of oxy-Hb and deoxy-Hb. Given that the magnitude and spatial location of the blood flow changes are tightly linked to changes in neural activity, a phenomenon known as neurovascular coupling, increases in oxy-Hb and decreases in deoxy-Hb are considered to reflect neural activity [14,15].

Compared to another neuroimaging technique, functional magnetic resonance imaging (fMRI), its low-cost and easy-to-maintain characteristics make fNIRS more accessible to researchers outside of the medical field. Its portable (in some models), less noisy, and less bodily constraining characteristics have made it the most common technique used by researchers to investigate the “online” brain activity during contact with nature. Employing fNIRS, previous studies have reported that viewing forest landscapes [16] or foliage plants [17]; viewing images of forests [18], garden landscapes [19], or a wooden interior wall [20,21]; smelling Japanese cypress leaf oil/air or Japanese cedar chips (for a review, [10]); touching a natural pothos leaf (Epipremnum aureum, [22]) or solid wood [23,24]; or transferring foliage plants [25] reduces the concentration of oxy-Hb in the prefrontal cortex (PFC). This has been interpreted as a calming or relaxing effect on the brain, which underlies many of the affective benefits or mood-improving effects achieved by contact with nature.

Furthermore, both fNIRS and fMRI have been used to study the “offline” or “after” brain activity following the end of contact with nature. Park et al. [26] reported that a 20-minute walk in the forest increased the subjects’ feelings of comfort and calmness compared to a similar walk in city areas. This was accompanied by a decrease in oxy-Hb concentration in the PFC as measured by fNIRS. Bratman et al. [27] found that a 90-minute walk in a natural environment with open grasslands and birds decreased self-reported rumination, a maladaptive self-reflective process that has been linked to depression. This decrease was not observed after a similar walk in an urban environment with steady traffic. Meanwhile, only the walk in nature decreased the activity of the subgenual PFC and the perigenual anterior cingulate cortex, two brain regions implicated in rumination and affective control (for a more extensive discussion, see [11]).

Taken together, the studies using fNIRS and fMRI so far have greatly advanced our understanding of the neural substrate of the mood-improving effects of contact with nature and have specifically identified a prominent role of the PFC. However, which specific regions within the PFC underlie this benefit and serve as the online neural substrates in particular remain unclear. fMRI is considered inappropriate for investigating the sensitive affective benefits of contact with nature and the online neural substrates due to its heavy noise and severe constraint on the subjects. fNIRS is more appropriate, however, previous studies have generally used models of fNIRS that provide only a single measurement for the broad area of the PFC (e.g., 2-channel NIRS). Therefore, in the present study, we aimed to identify the specific regions involved in the mood-improving effect of viewing images of nature using a 52-channel NIRS instrument (ETG-4000; Hitachi Medical Co., Tokyo, Japan).

This ETG-4000 instrument measures the activity of the frontal area with a maximum of 52 channels, the locations of which have been anatomically confirmed in the standard brain space [28]. This instrument has been widely used in the field of psychiatry (e.g., reports from our department, [29,30]). In fact, when combined with the verbal fluency test, this instrument has been employed as an *advanced medical technique* (a Japanese governmental medical system) during 2009–2014 and then included in national health insurance since 2014 by the Japanese government for aiding the differential diagnosis of depressive states (mainly based on the findings of [31]). In the present study using this instrument, we focused on the orbitofrontal cortex (OFC) and dorsolateral prefrontal cortex (dlPFC), two regions of the PFC that have been consistently implicated in affective processing and control [32,33,34,35]. We predicted that the mood-improving effect of viewing images of nature is accompanied by changes in the activity of these two regions. Specifically, we predicted medium effect sizes based on a previous meta-analysis [2] that reported an average effect size of r = 0.26 (equivalent to Cohen’s *d* = 0.54) for the effect of nature pictures on positive and negative affect.

## 2. Materials and Methods

### 2.1. Participants

The study was approved by the Institutional Review Board of Yamaguchi University Hospital and preregistered on the University hospital Medical Information Network Clinical Trial Registry (UMIN-CTR, register ID: UMIN000041123). Thirty subjects (21 males and 9 females, with an age of 23.20 ± 1.94 years) were recruited via posters placed on campus and the department’s homepage, and through word-of-mouth in Yamaguchi, Japan during the period from July to November 2020. The study was carried out in accordance with the latest version of the Declaration of Helsinki. All subjects agreed to participate in this study and provided written informed consent after receiving a detailed explanation of the study. The inclusion criteria were (1) being 20–29 years old at the time of the visit and (2) having minimum eyesight of 0.6 (with correction, based on self-report). The exclusion criteria were (1) having self-reported color-blindness, (2) currently suffering from a mental illness or undergoing medical examination based on self-report, (3) being a staff member of our department who receives personnel evaluation directly by the principal investigator of this study, and (4) being judged to be unsuitable for this study (e.g., having attended our pilot study that aimed to confirm the mood-improving effect of nature images). No participant was excluded due to meeting any of the exclusion criteria.

### 2.2. Design and Procedure

Subjects were asked to follow three instructions before participating in this study: (1) get enough sleep the night before, (2) refrain from drinking coffee and energy drinks, smoking, and engaging in intensive physical activity in the two hours prior to visiting the laboratory, and (3) contact the staff and rescheduling the experiment if they were sick or did not feel well on the day of the experiment (e.g., because of menstrual pain for females). On the day of the experiment, the subjects first filled out a questionnaire to answer their age, gender, history of smoking and alcohol intake, education, handedness, and to confirm whether they had adhered to the above instructions.

**Figure 1:**
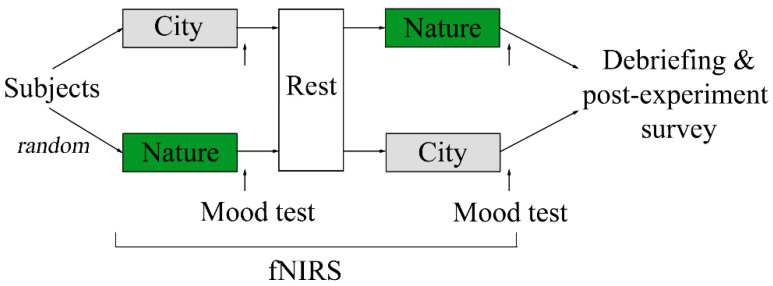
Schematic illustration of the study design.

In this study, we used a randomized controlled crossover design and assigned subjects to view images of nature and built environments (hereafter city) in a counterbalanced order (Figure 1). Thirty subjects were randomly assigned to one of two image viewing orders: (1) city → nature or (2) nature → city. Each image viewing session lasted three minutes. Immediately after each image viewing session, the subjects were instructed to indicate their mood at the moment in terms of comfortableness, relaxation, and vigor using a visual analog scale (VAS). After the first image viewing session and mood test, the subjects rested for three minutes as a wash-out period. During the two image viewing sessions, subjects wore a fNIRS probe cap (ETG-4000; Hitachi Medical Co., Tokyo, Japan) on their head, which measured the concentration of oxy-Hb, and an Apple Watch Series 4 (Apple Inc., Los Altos, CA, USA) on the non-dominant wrist, which measured their heart rate [36]. Of the 30 subjects who agreed to participate in this study, five were excluded due to improper placing of the fNIRS probe cap (e.g., thick hair) or reporting feelings of sleepiness during the experiment. This left 25 subjects (16 males, 9 females) for our final analysis, with an age of 23.04 ± 1.67 years, all of whom were right-handed.

### 2.3. Stimuli

**Figure 2:**
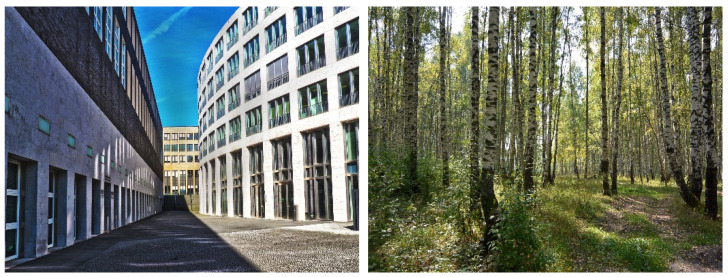
Example images of city and nature.

The visual images of city and nature were presented via a 27-inch high resolution monitor (Dell S2718H, Dell Inc., Round Rock, Texas, USA) using MATLAB R2018b (The MathWorks, Inc., Natick, MA, USA) and Psychtoolbox 3 [37]. For each image category, 12 images were presented, and each image was shown for 15 seconds (adding up to three minutes in total). The images of city featured buildings, while those of nature featured green plants (for example images, Figure 2). Subjects were instructed to vividly imagine the scenery in the images actually spreading out in front of them.

### 2.4. Mood Test

Immediately following each image viewing session, the subjects were instructed to indicate their mood at that moment in terms of comfortableness, relaxation, and vigor using a VAS. This mood test was designed based on the valence–arousal two-dimensional affect grid [38] and consisted of three pairs of adjectives at the two extreme ends of the scale, *uncomfortable*-*comfortable*, *tense*-*relaxed*, and *mentally tired*-*vigorous*. The VAS had a length of 100 mm, and subjects were asked to place a “×” mark on the scale that best fits their experience at that moment. The distance of the mark from the left side of the scale was measured (in mm) and used as the mood score. The continuous aspect of the VAS makes it superior to discrete scales such as the Likert scale in detecting small changes [39,40,41,42].

### 2.5. FNIRS

**Figure 3:**
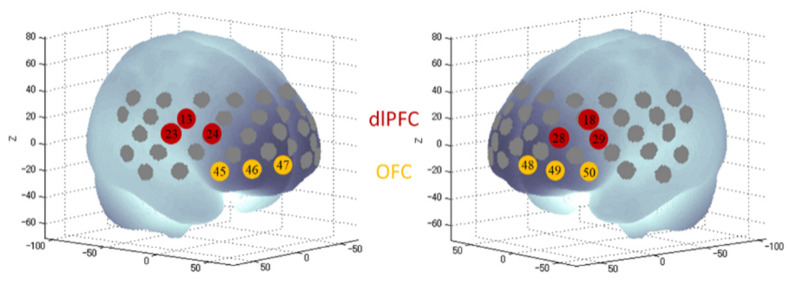
Regions of interest defined by channels in fNIRS. Projection of the NIRS channels on the cortical surface based on a MNI-152 compatible canonical brain [46].

We used the 52-channel NIRS instrument ETG-4000 (Hitachi Medical Co., Tokyo, Japan) and followed the standard procedure of fNIRS measurement [29,30,43]. Specifically, a multichannel probe holder (3 × 11), consisting of 17 eliminating and 16 detecting probes resulting in 52 channels, was placed in accordance with the international 10–20 system, with the lowest probes being positioned along the T3-Fp1-Fp2-T4 line. The probes’ arrangement allowed for the measurement of the bilateral PFC, ventro-lateral, fronto-polar, and part of the temporal cortical surface area. For the present study, we defined four regions of interest (ROIs, Figure 3): the right dlPFC (channels 13, 23, 24), the left dlPFC (channels 18, 28, 29), the right OFC (45, 46, 47), and the left OFC (48, 49, 50), based on the anatomical labeling by Okamoto et al. [28] and adoption by previous studies [44,45]. After the fNIRS probe cap placement, a probe check was conducted to examine the signal intensity of each channel. We confirmed that all the four ROIs’ channels had adequate signal intensity. In cases where any of the channels had inadequate signal intensity, probe adjustments were performed.

The time resolution of the measurement was 0.1 seconds. Following previous studies in this field [10], the concentration of oxy-Hb was used as the primary indicator of brain activity. Data were analyzed using the “continuous mode”, and channels with low signal-to-noise ratios and motion artifacts were deleted. Of the three channels of a ROI, if one or two error channels existed, they were deleted, and the remaining channel(s) were used for the calculation of the ROI average. If all three channels of a ROI for one subject were deleted, the ROI of that subject was removed from further analysis. Finally, to compensate for the effect of baseline drift over time and reduce the residual/carryover effect of the previous intervention (for the second image viewing session), we conducted a baseline correction of the concentration of oxy-Hb for each channel, in which the mean value of the baseline (10 seconds preceding image viewing) was subtracted from each time point.

### 2.6. Blindness and Debriefing

To exclude the mood-improving expectancy of viewing images of nature, subjects were blinded about the experiment’s true objective and were instead informed that they were participating in a study titled *The Effect of Daily Activities on the Brain*. With regard to the intervention assignment, they were told that there were two kinds of intervention, image viewing and light physical activities, and they were assigned to the image viewing group according to random allocation. With regard to the second session of intervention, they were informed that it was for the purpose of confirmation with a different category of images. After the experiment, subjects were debriefed and questioned (i.e., post-experiment survey) about to what extent they noticed the real purpose of the study (i.e., investigation of the mood-improving effect of viewing images of nature vs. city) during the experiment.

The experimenters of the study were blind in such a way that they were unaware of the intervention order (i.e., city → nature or nature → city) until the start of the first image viewing. The experimenters that performed data analysis were also unaware of the intervention order during data analysis.

### 2.7. Statistical Analysis

The statistical analysis was conducted with IBM SPSS Statistics 26.0 (IBM Corp. in Armonk, NY, USA) and MATLAB R2018b (The MathWorks, Inc., Natick, MA, USA). Paired *t*-tests were used to compare the differences in mood, oxy-Hb concentration, and heart rate between viewing images of nature vs. city. Repeated measures ANOVAs were used to control the covariate of the post-experiment survey (i.e., whether or not they noticed the real purpose of the study during the experiment). Effect size (Cohen’s *d*) was calculated for all between-group comparisons. A significance level of *p* < 0.05 was used.

## 3. Results

### 3.1. Mood Measures

**Figure 4:**
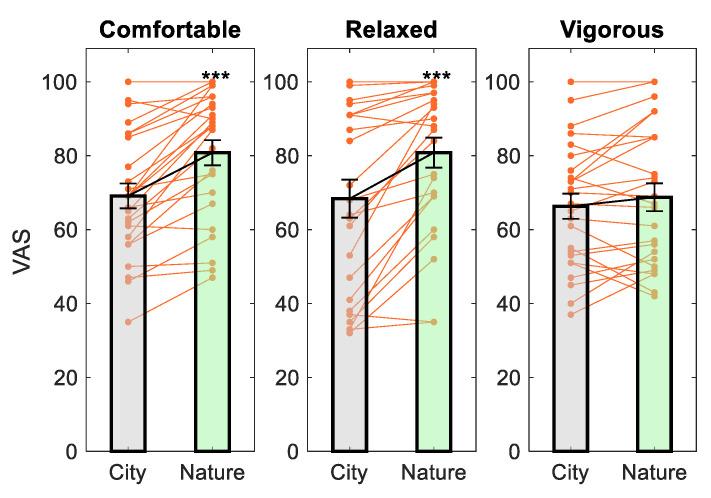
The effect of image viewing on mood. *** *p* < 0.001 compared to City.

Paired *t*-tests indicated that subjects reported significantly greater feelings of comfortableness (*t*(24) = −6.21, *p* = 2 × 10^−6^, *d* = 1.24) and relaxation (*t*(24) = −4.78, *p* = 7 × 10^−5^, *d* = 0.96) but not vigor (*t*(24) = −1.47, *p* = 0.154, *d* = 0.29) after viewing images of nature compared to city. These effect sizes were considered large. The results are plotted in Figure 4.

### 3.2. Brain Activity

**Figure 5:**
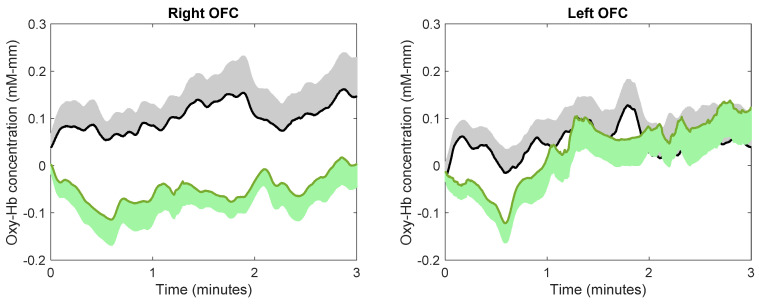

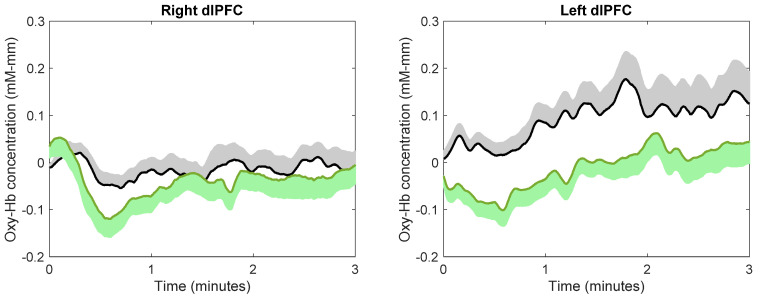
The effect of image viewing on brain activity. Green and black lines indicate the mean oxy-Hb concentration while viewing images of nature and city, respectively; green and black shadows indicate the standard error.

The continuous changes in the concentration of oxy-Hb during image viewing for each ROI are shown in Figure 5. Using the average concentration of oxy-Hb across the three minutes of image viewing, paired t-tests indicated that subjects showed a significantly lower concentration of oxy-Hb while viewing images of nature compared to city in the right OFC (*t*(23) = 2.81, *p* = 0.0098, *d* = 0.57). This effect size was considered medium. There was no difference in the average concentration of oxy-Hb between viewing images of nature and city in the left OFC (*t*(24) = 0.183, *p* = 0.857, *d* = 0.036), right dlPFC (*t*(22) = 0.669, *p* = 0.511, *d* = 0.14), or left dlPFC (*t*(23) = 1.9430, *p* = 0.064, *d* = 0.38).

### 3.3. Heart Rate

**Figure 6:**
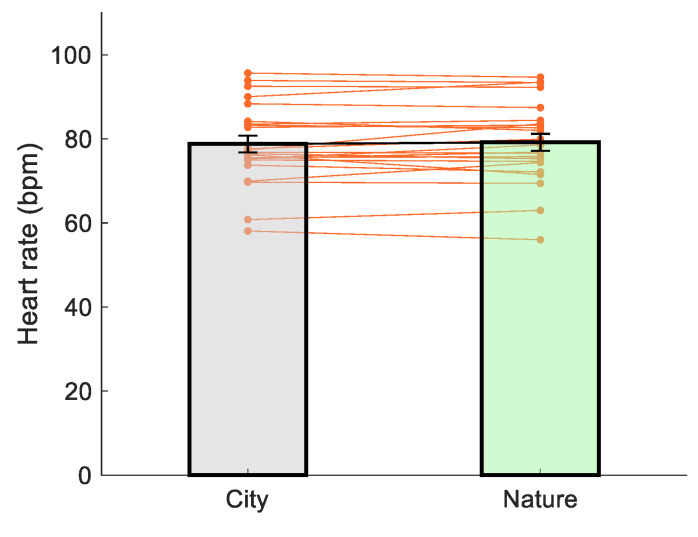
The effect of image viewing on heart rate.

There was no difference in heart rate over the three minutes between viewing images of nature vs. city (*t*(22) = −0.806, *p* = 0.429, *d* = 0.17, Figure 6).

### 3.4. Post-Experiment Survey

All of the above between-group differences remained significant after incorporating whether subjects noticed the experiment’s true objective as a covariate (repeated measures ANOVAs). Specifically, the statistics for comfortableness were F_1,23_ = 12.979, *p* = 0.002; relaxation, F_1,23_ = 10.055, *p* = 0.004; and oxy-Hb in the right OFC, F_1,22_ = 6.071, *p* = 0.022.

## 4. Discussion

In the present study, we confirmed the mood-improving effect of viewing images of nature with a sample of young adults: compared to viewing images of city, viewing images of nature increased subjects’ feelings of comfortableness and relaxation with a large effect size. Furthermore, we found that this mood-improving effect was accompanied by reduced activity (as indicated by oxy-Hb concentration) in the right OFC (with a medium effect size). Consistent with previous theories [9,10,11,12], these results indicate the possibility that viewing images of nature might exert its mood-improving effect via calming the right OFC. Importantly, these results remained unchanged even after controlling whether the subjects noticed the true objective of the study. This suggests that both incidental and intentional exposure to nature may have a mood-improving effect.

It is not immediately clear why the involvement of the right but not left OFC is identified here. A careful review of our previous NIRS studies of contact with nature (Appendix A) shows that whereas 53% of the studies (10/19) found similarly reduced activity in both hemispheres of the PFC, 32% of the studies (6/19) found reduced activity in the right PFC only, with another 11% of the studies (2/19) finding reduced activity in the left PFC only. In contrast, one study (5%) found that the activity of either hemisphere of the PFC may decrease depending on the stimuli: viewing smooth wood reduces the activity of the left PFC, while viewing knotty wood reduces the activity of the right PFC. It remains for future studies to clarify the precise role of the two hemispheres in the calming effect of contact with nature, but one observation that may deserve consideration is that the right hemisphere is more specialized in processing nonverbal (e.g., pictorial or image-related) information [47,48].

Although we predicted that both the OFC and the dlPFC are involved in the mood-improving effect of viewing images of nature, only the involvement of the OFC is confirmed here. The OFC is well-known for playing an essential role in value representation and affective processing through integrating information from sensory cortices, brain stem autonomic areas, and the amygdala [32,33]. The OFC has reciprocal projections with the amygdala, another area important for evaluating emotionally salient (in particular aversive, unpleasant) information [49]. The reduced activity in the right OFC in the present study might reflect reduced activity in the amygdala, which has been identified after contact with nature in fMRI studies (for a more extensive discussion, see [11]). Notably, the OFC has been implicated in the pathophysiology of depression and anxiety [50,51]. Patients with depression [50] and anxiety [51] demonstrate increased activity in the OFC at rest, which is attenuated after pharmacological treatment. The abnormally increased activity in the OFC is considered to be a result of a hyperactive amygdala in these patients. Thus, one may speculate that viewing images of nature or other means of contact with nature might have therapeutic effects for these patients by modulating the activity in the OFC (and potentially the amygdala). It deserves future research to test this speculation with patients.

Meanwhile, we cannot conclude that the dlPFC is not involved in the mood-improving effect of contact with nature, given that we observed reduced activity in the left dlPFC with a trend towards significance (*p* = 0.064). Future investigations may specifically target the dlPFC and examine whether it is affected by contact with nature.

An important issue we noticed while conducting the current research was that the field has focused too much on significance while ignoring the necessity of evaluating effect sizes [52]. We hope the current study, via reporting effects sizes for all between-group comparisons, may call attention of researchers in this field. By examining, interpreting, and applying effect sizes more carefully, we hope future research will propel us towards an advanced understanding of the mood-improving effect of contact with nature.

Several limitations of our study should be noted. First, we only investigated the immediate effect of viewing images of nature. Whether the effect quickly fades and how long it lasts remains to be investigated. One previous study showed that the physiological effect of a one-day forest therapy session on blood pressure lasted for at least five days [53]. Few people will expect that a 3-minute image viewing improves mood and calms the brain for days; however, investigating exactly how long the effect lasts may be informative for people to practice nature therapy in their daily lives. Second, although we used an Apple Watch Series 4 (Apple Inc.) to monitor heart rate, we were unable to record the heart rate variability data, and therefore could not provide an evaluation of autonomic nervous activity that has been frequently employed in previous studies [10,12,21]. Third, subjects in their twenties were examined in the present study. Future research with more diverse samples is required to investigate whether gender and age differences exist in the findings we reported. Lastly, we employed images featuring green plants. Future research may investigate the neural substrate underlying the mood-improving effect of contact with nature of other colors, such as fresh red roses [54] and “blue spaces” containing aquatic elements (e.g., oceans and rivers; [55,56]).

Despite these limitations, the present study has important implications for our daily lives. For instance, we may intentionally increase our exposure to nature or build a nature-rich environment around us to increase our incidental exposure. This can simply be the addition of indoor plants, nature posters, or using nature images as computer, tablet, and smartphone wallpapers. This can also be outdoor activities in the natural environment, such as having a walk in the park. These kinds of daily activities may help calm our brain and improve our mood.

## 5. Conclusions

In a randomized controlled crossover trial with a small sample of young adults, we found that viewing images of nature increased comfortableness and relaxation compared to viewing images of built environments with a large effect size. This was accompanied by decreased activity in the right OFC while viewing images of nature compared to built environments, with a medium effect size. Therefore, viewing images of nature might improve mood by reducing the activity of or calming the right OFC. Our findings may have important implications for patients with depression and anxiety since their OFC is reported to be hyperactive at rest. Future research may investigate whether contact with nature has therapeutic effects for these patients, via, for instance, calming the OFC.

## Data Availability

The data that support the findings of this study are available from the corresponding author upon reasonable request.

## Appendix A

A summary of the studies from The Nature Therapy Project conducted at the Center for Environment, Health and Field Sciences, Chiba University. For Appendix details, please see the attached Excel file.

These studies were selected based on the following criteria: (1) they used a 2-channel NIRS or time-resolved spectroscopy (TRS) to measure the concentration of oxyhemoglobin (oxy-Hb) in the prefrontal cortex (PFC), (2) they observed a statistically significant difference in the concentration of oxy-Hb in at least one hemisphere of the PFC based on the comparison between Stimulation and Control/Baseline, and (3) they observed a decrease in the concentration of oxy-Hb from the baseline for the nature-related stimulation.
